# Typhoid Fever among Children, Ghana

**DOI:** 10.3201/eid1611.100388

**Published:** 2010-11

**Authors:** Florian Marks, Yaw Adu-Sarkodie, Frank Hünger, Nimako Sarpong, Samuel Ekuban, Alex Agyekum, Bernard Nkrumah, Norbert G. Schwarz, Michael O. Favorov, Christian G. Meyer, Jürgen May

**Affiliations:** Author affiliations: International Vaccine Institute, Seoul, South Korea (F. Marks, M.O. Favorov);; Kwame Nkrumah University of Science and Technology, Kumasi, Ghana (Y. Adu-Sarkodie);; Kumasi Centre for Collaborative Research in Tropical Medicine, Kumasi (F. Hünger, N. Sarpong, S. Ekuban, A. Agyekum, B. Nkrumah);; Bernhard Nocht Institute for Tropical Medicine, Hamburg, Germany (N.G. Schwarz, C.G. Meyer, J. May)

**Keywords:** Typhoid fever, Ghana, children, Salmonella, bacteria, letter

**To the Editor:** Typhoid fever (TF) remains a problem of concern in many low-income countries. *Salmonella enterica* serovar Typhi causes ≈22,000,000 symptomatic infections and 220,000 fatalities worldwide annually (*1*). However, the effect and incidence of TF in many parts of sub-Saharan Africa are largely unknown because diagnostic laboratories are lacking and fatal TF is frequently attributed to malaria (*2*,*3*). In Ghana, TF ranks among the leading 20 causes of outpatient illness, accounting for 0.92% of hospital admissions (*4*).

We conducted our study at the rural Agogo Presbyterian Hospital in the Ashanti Region of Ghana. The percentage of residents of 99 villages and household clusters of buildings (population size 18–13,559 persons, median 277 persons) with access to the study hospital was assessed in a healthcare utilization survey. A proportional-to-size number of children were randomly selected in each village, and a standardized interview was conducted. TF incidences were calculated for September 2007–November 2008 (Table). A bacteriology laboratory with BACTEC 9050 automated blood culture system (Becton Dickinson, Sparks, MD, USA) was established in the study hospital and run to assess the number of admissions with TF, the incidence of TF in the adjoining community and *S. enterica* ser. Typhi resistance to a panel of antimicrobial drugs.

**Table:** Estimates of *Salmonella* *enterica* serovar Typhi incidence in children, Ghana, September 2007–November 2008

Age group, y	*No. S. enterica* ser. Typhi isolates*	Coverage population†	Incidence‡ (95% confidence interval)
0–15	16	14,933	120 (70 to 170)
<2	1	2,133	50 (–30 to 140)
2–<5	8	3,200	290 (120 to 450)
5–<8	5	2,880	200 (50 to 340)
8–<11	1	2,880	40 (–30 to 110)
11–<15	1	3,840	30 (–20 to 80)

*Observation period 15 mo (period of complete and uninterrupted assessment of blood culture data). †No. residents of each community with access to Agogo Presbyterian Hospital. ‡Per 100,000 persons per year.

The study included 1,456 children <15 years of age who were admitted to the pediatric ward of Agogo Presbyterian Hospital over the 23-month study period. Overall, 52.1% were male; mean age of children was 32.2 months (SD ± 36.0 months; median 19 months, range 0–174 months). Blood was cultured by using a BACTEC 9050 blood culture system (Becton Dickinson), and positive samples were examined by standard methods. Antimicrobial drug susceptibility testing was performed on all serovar Typhi isolates by using the Kirby-Bauer disk-diffusion method for ampicillin, chloramphenicol, tetracycline, trimethoprim/sulfamethoxazole, amoxicillin/clavulanic acid, gentamicin, ciprofloxacin, and ceftriaxone.

Children <2 years of age had the highest proportion of positive blood cultures (164/1,456, 21.3%; Figure A1). Of 298 blood cultures yielding positive growth for bacterial pathogens or for *Candida* spp., 37 (12.4%) isolates (2.5% of the 1,456 hospitalized children) were positive for *S. enterica* ser. Typhi. The frequency of TF was low among children <2 years of age (7/1,018, 0.7%), increased among those 2 to <11 years of age (29/417, 7.0%), and decreased among children ≥11 years of age (1/22, 4.6%) (Figure A1). One (2.7%) child with TF died. Malaria parasites were detected in 2 children with *S. enterica* ser. Typhi. Pathogens other than *S. enterica* ser. Typhi were identified among 21.3% and 11.8% of children 0 to <2 years and 5 to <8 years of age, respectively. These pathogens included nontyphoidal salmonellae, *Staphylococcus aureus*, and *Streptococcus pneumoniae*. *S. enterica* ser. Typhi isolates were resistant to chloramphenicol (73%), trimethoprim/sulfamethoxazole (71%), ampicillin/amoxicillin (70%), tetracycline (64%), gentamicin (46%), and amoxicillin/clavulanic acid (24%) but susceptible to ciprofloxacin and ceftriaxone.

TF incidence in children <5 years of age was ≈190 cases/100,000 population and highest in children 2–5 years of age (290/100,000 per year) and 5–8 years of age (200/100,000 per year) (Table). In children older than 8, incidence decreased continuously, and the number of cases was too low to enable precise age-stratified incidence calculations. The incidences in the study area point to a higher impact of TF than expected (*4*) and may reflect an underestimation of TF in other West African regions as well. Our high incidence figure may still underestimate the incidence because of a low sensitivity of standard microbiologic methods (up to 50%), which are prone to underdiagnose moderate bacteremia in *Salmonella* infections (*5*,*6*).

Compared with Asia, only limited data are available from Africa on *S. enterica* ser. Typhi drug resistance. A study from Nigeria showed that, among serovar Typhi strains isolated from hospitalized patients in Lagos during 1997–2004, resistance rates reached 87% for ampicillin and were 0.7% for ciprofloxacin, compared with 70% and 0%, respectively, in the present study. Resistance to trimethoprim/sulfamethoxazole was 59% in Nigeria, compared with 71% in Ghana. In Togo, proportions of serovar Typhi strains resistant to chloramphenicol and trimethoprim/sulfamethoxazole were 33% and 46%, respectively, before 2002 and 73% and 79% in 2003–2004 (*7*) and thus similar to those in our study.

In addition, resistances to ciprofloxacin and ceftriaxone were <10%. Multidrug resistance (resistance to ampicillin, trimethoprim/sulfamethoxazole, and chloramphenicol) was observed in 63% of children in our study, compared with 7% in India, 22% in Vietnam, and 65% in Pakistan (*8*–*10*).

More effort is needed in Africa to enable reliable and standardized laboratory diagnoses of *Salmonella* infections and to sustain TF surveillance and drug sensitivity surveys. Moreover, introduction of a vaccination program should be discussed after more data are obtained from other areas in Ghana and West Africa. Such data currently are collected in an extensive standardized surveillance program across the continent performed by our group and others. In parallel, trials should be conducted to assess the effectiveness and cost-effectiveness of currently available and newly developed TF vaccines.

## Figures and Tables

**Figure A1:**
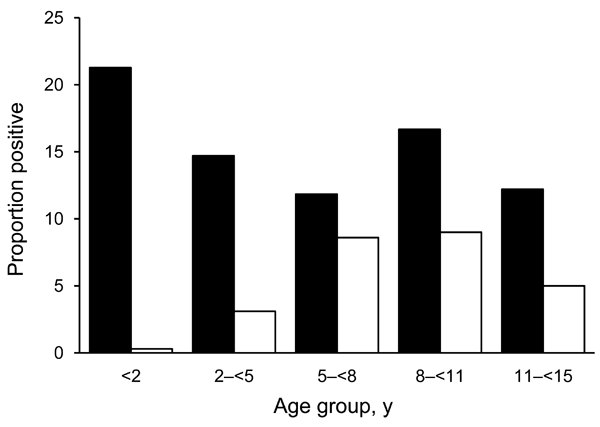
Age-stratified proportion of blood cultures positive for any bacteria without the exception of contaminants (black columns) and of blood cultures positive for *Salmonella enterica* serovar Typhi (white columns); denominator is the total number of blood cultures performed. Age groups and numbers of hospitalized children who had blood cultured were as follows: <2 years (771 children), 2 to <5 years (415), 5 to <8 years (152), 8 to <11 years (78), and 11 to <15 years (40).
